# A local anesthetic, ropivacaine, suppresses activated microglia via a nerve growth factor-dependent mechanism and astrocytes via a nerve growth factor-independent mechanism in neuropathic pain

**DOI:** 10.1186/1744-8069-7-2

**Published:** 2011-01-07

**Authors:** Shigeru Toda, Atsushi Sakai, Yumiko Ikeda, Atsuhiro Sakamoto, Hidenori Suzuki

**Affiliations:** 1 Department of Anesthesiology, Nippon Medical School, 1-1-5 Sendagi, Bunkyo-ku, Tokyo 113-8602, Japan; 2 Department of Pharmacology, Nippon Medical School, 1-1-5 Sendagi, Bunkyo-ku, Tokyo 113-8602, Japan

## Abstract

**Background:**

Local anesthetics alleviate neuropathic pain in some cases in clinical practice, and exhibit longer durations of action than those predicted on the basis of the pharmacokinetics of their blocking effects on voltage-dependent sodium channels. Therefore, local anesthetics may contribute to additional mechanisms for reversal of the sensitization of nociceptive pathways that occurs in the neuropathic pain state. In recent years, spinal glial cells, microglia and astrocytes, have been shown to play critical roles in neuropathic pain, but their participation in the analgesic effects of local anesthetics remains largely unknown.

**Results:**

Repetitive epidural administration of ropivacaine reduced the hyperalgesia induced by chronic constrictive injury of the sciatic nerve. Concomitantly with this analgesia, ropivacaine suppressed the increases in the immunoreactivities of CD11b and glial fibrillary acidic protein in the dorsal spinal cord, as markers of activated microglia and astrocytes, respectively. In addition, epidural administration of a TrkA-IgG fusion protein that blocks the action of nerve growth factor (NGF), which was upregulated by ropivacaine in the dorsal root ganglion, prevented the inhibitory effect of ropivacaine on microglia, but not astrocytes. The blockade of NGF action also abolished the analgesic effect of ropivacaine on neuropathic pain.

**Conclusions:**

Ropivacaine provides prolonged analgesia possibly by suppressing microglial activation in an NGF-dependent manner and astrocyte activation in an NGF-independent manner in the dorsal spinal cord. Local anesthetics, including ropivacaine, may represent a new approach for glial cell inhibition and, therefore, therapeutic strategies for neuropathic pain.

## Background

Neuropathic pain is an extremely severe chronic pain caused by damage to the nervous system itself. In clinical practice, this pain syndrome remains a major issue because of the limited and variable effectiveness of existing analgesics [1]. Such poor effectiveness can be partly attributed to insufficient understanding of the analgesic mechanisms of existing drugs such as opioids, anticonvulsants and antidepressants. Systemic and epidural applications of local anesthetics relieve neuropathic pain in some cases, but the underlying mechanisms remain unclear because the effects occur at lower doses than the effective doses for blocking voltage-gated sodium channels, the primary targets of local anesthetics [2]. Furthermore, the analgesic effects of drugs persist for longer durations than those predicted on the basis of their pharmacokinetics [3-5]. It has been reported that local anesthetics affect other ion channels and G protein-coupled receptors [2], and these effects are suggested to partially contribute to the analgesia observed with local anesthetics [2,6]. Local anesthetics also inhibit the phosphorylation of p38 mitogen-activated protein kinase in the spinal microglia in animal models of neuropathic pain [7,8]. However, the glial participation in the analgesic effects of local anesthetics remains speculative.

Microglia and astrocytes are the dominant glial cells in the central nervous system and play critical roles in neuroinflammation and neuronal plasticity via active communication with neurons [9,10]. In response to peripheral nerve injury, these glial cells become activated and release a variety of proinflammatory mediators such as cytokines and chemokines in the dorsal spinal cord [11-14]. These proinflammatory mediators act on nociceptive neurons, resulting in augmentation of nociceptive signal transmission or central sensitization. In line with these findings, it has been reported that intrathecal administration of compounds that suppress the activation of glial cells or antagonists of proinflammatory mediators alleviates neuropathic pain [11-13,15-18]. Therefore, modulating glial cell function appears to represent a promising therapeutic strategy for neuropathic pain.

Nerve growth factor (NGF) is a founding member of the neurotrophic factor family and is well known to be involved in nociceptor function [19]. In the periphery, NGF is released in response to inflammation and subsequently acts on its high-affinity receptor, TrkA, expressed on a subset of nociceptive dorsal root ganglion (DRG) neurons, resulting in hyperalgesia [19]. On the other hand, NGF has a beneficial impact on neuropathic pain when administered intrathecally [20,21]. NGF promotes functional regeneration of damaged DRG neurons [22] and improves neuropathy in streptozotocin-induced diabetic rats [23]. NGF ameliorates the increased expressions of c-jun and ATF3 in DRG neurons caused by nerve injury, suggesting its involvement in the protection of neurons [24,25]. It has also been reported that NGF suppresses activated astrocytes in association with pain relief [26]. Thus, the roles of NGF in neuropathy are considerably complicated.

Ropivacaine was developed as an alternative to bupivacaine, which has more severe toxicity, and at present it is widely used as an epidural anesthetic at concentrations of 0.2-1% in clinical practice [27]. Ropivacaine shows increased cardiovascular safety and a shorter elimination half-life compared with bupivacaine [27-29]. Recently, we reported that the content of NGF was upregulated in the injured DRG after repetitive epidural administration of 0.2% ropivacaine [30]. This finding implies that NGF produced endogenously upon ropivacaine treatment plays a role in the process of pain reduction. Therefore, in the present study, we further investigated the involvement of NGF in the analgesic effect of ropivacaine in a rat model of neuropathic pain with a focus on the spinal glial cells.

## Results

### Prolonged analgesic effect of repetitive epidural administration of ropivacaine on neuropathic pain

**Figure 1:**
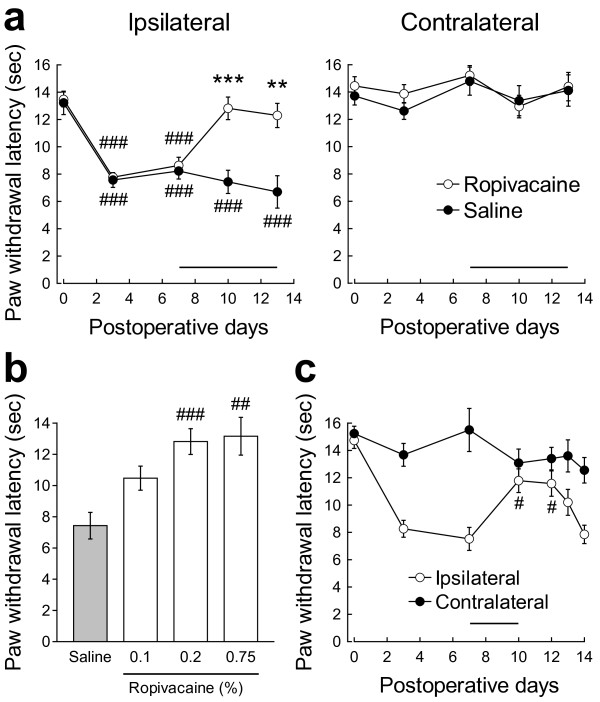
**Effect of epidural ropivacaine treatment on thermal hyperalgesia in CCI rats**. (a) The latencies for paw withdrawal on the ipsilateral (left panel) and contralateral (right panel) sides in response to thermal stimuli applied to the corresponding hindpaw pad in rats with CCI were evaluated. Ropivacaine (open circles) or saline (closed circles) was epidurally administered once daily from days 7 to 13, as indicated by the horizontal bars. **p < 0.01 and ***p < 0.001 compared with the values in CCI rats treated with saline on the corresponding days by an unpaired *t*-test (*n *= 8-9). ^###^*p *< 0.001 compared with the values on the preoperative day by Dunnett's multiple comparison test (*n *= 8-9). (b) The dose-dependent effects of epidural ropivacaine on the thermal hyperalgesia were examined at day 10. ^##^*p *< 0.01 and ^###^*p *< 0.001 compared with the values in CCI rats treated with saline by Dunnett's multiple comparison test (*n *= 4-9). (c) The duration of analgesia induced by ropivacaine was examined. Ropivacaine was epidurally administered once daily from days 7 to 10, as indicated by the horizontal bars. ^#^*p *< 0.05 compared with the values before ropivacaine administration at day 7 by Dunnett's multiple comparison test (*n *= 4).

Before the chronic constrictive injury (CCI) operation on day 0, the latencies of ipsilateral paw withdrawal from thermal stimulation in rats assigned to a ropivacaine treatment group and a saline treatment group were 13.4 ± 0.5 s and 13.2 ± 0.9 s, respectively (*n *= 8-9; Figure 1a). After the CCI operation, the latencies began to decrease significantly on the ipsilateral side from day 3 (7.8 ± 0.3 s for ropivacaine treatment and 7.6 ± 0.5 s for saline treatment; *p *< 0.001 for both treatments; Figure 1a). The latencies on the contralateral side were unchanged (Figure 1a).

Next, we examined the effect of repetitive epidural administration of 0.2% ropivacaine on the thermal hyperalgesia in CCI rats. Epidural administration of ropivacaine or saline was commenced at day 7 after the CCI operation, when the hyperalgesia had become established, and was continued once daily for 7 days. Motor paralysis was observed for about 10 min immediately after the epidural injection of ropivacaine, but the paralysis was limited to the hindlimbs in all of the rats examined, as described by Durant and Yaksh [31]. Such behavioral changes confirmed that ropivacaine had been successfully delivered to the epidural space around the lumbar third (L3) vertebral level and suggested that ropivacaine at least transiently inhibited the voltage-gated sodium channels in this protocol. The thermal hyperalgesia of CCI rats was significantly relieved at 3 days after the beginning of ropivacaine treatment compared with CCI rats administered saline (12.8 ± 0.8 s for ropivacaine treatment and 7.4 ± 0.9 s for saline treatment; *p *< 0.001; *n *= 8-9; Figure 1a). The thermal hyperalgesia had almost returned to the basal level at day 13 (13.5 ± 0.5 s before CCI and 12.3 ± 0.9 s after CCI plus ropivacaine). Treatment with ropivacaine and saline did not affect the contralateral paw withdrawal latencies in response to the thermal stimulus (14.4 ± 1.0 s for ropivacaine treatment and 14.1 ± 1.1 s for saline treatment at day 13; *n *= 8-9; Figure 1a). The analgesic effects of other concentrations of ropivacaine were also examined. Ropivacaine reduced the thermal hyperalgesia in a dose-dependent manner in the concentration range of 0.1-0.75% at day 10 (*n *= 4-9; Figure 1b).

Furthermore, we investigated the duration of analgesia induced by repetitive epidural ropivacaine administration. Ropivacaine administration to CCI rats was commenced from day 7, but discontinued after the administration at day 10, at which time point the recovery from the hyperalgesia was established by the repeated ropivacaine injections. After the cessation of ropivacaine administration, the significant reduction of the thermal hyperalgesia persisted for at least 2 days (7.5 ± 0.8 s at day 7 before ropivacaine administration and 11.6 ± 0.9 s at day 12; *p *< 0.05; *n *= 4; Figure 1c), and then the analgesic effect gradually declined (Figure 1c).

### Suppression of activated spinal glial cells by ropivacaine administration

**Figure 2:**
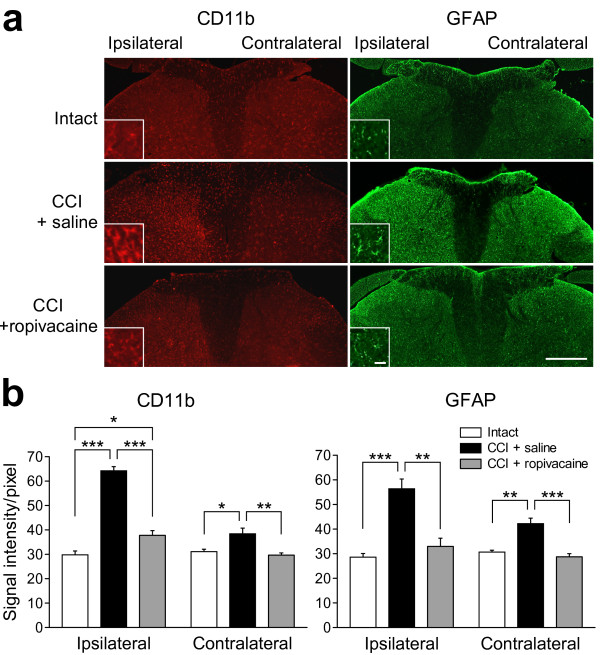
**Effects of epidural ropivacaine treatment on the activated spinal glial cells in CCI rats**. (a) Representative images of immunohistochemical staining for CD11b (a microglial activation marker) and GFAP (an astrocyte activation marker) in the L4 spinal dorsal horn. L4 spinal cords were obtained from control intact rats, CCI rats treated with saline and CCI rats treated with ropivacaine at day 14 after CCI. Scale bar, 200 μm. Insets: High-magnification views of immunoreactive cells. Scale bar, 5 μm. (b) Densitometric quantification of CD11b and GFAP immunoreactivities on the ipsilateral and contralateral sides. *p < 0.05, **p < 0.01 and ***p < 0.001 by Tukey-Kramer's multiple comparison test (*n *= 4).

To examine the involvement of spinal glial cells in the ropivacaine-induced analgesia, we analyzed the expressions of glial cell activation markers in the spinal dorsal horn of CCI rats. The immunoreactivity for CD11b, a microglial activation marker, was significantly increased in the ipsilateral spinal dorsal horn at day 14 after CCI (64.2 ± 1.7 a.u.) compared with control intact rats (29.8 ± 1.6 a.u.; *p *< 0.001; *n *= 4; Figure 2a and 2b). The immunoreactivity for glial fibrillary acidic protein (GFAP, an astrocyte activation marker) was also significantly increased on the ipsilateral side (56.3 ± 4 a.u. for CCI rats and 28.6 ± 1.5 a.u. for control intact rats; *p *< 0.001; *n *= 4; Figure 2a and 2b). The CD11b-expressing and GFAP-expressing cells changed their morphologies to amoeboid and hypertrophied with thick processes, respectively, indicating their activation after the CCI operation (Figure 2a). In ropivacaine-treated CCI rats, the immunoreactivity for CD11b was significantly decreased compared with saline-treated CCI rats (37.8 ± 1.9 a.u. for ropivacaine treatment and 64.2 ± 1.7 a.u. for saline treatment; *p *< 0.001; *n *= 4; Figure 2a and 2b). The immunoreactivity for GFAP was also decreased after ropivacaine treatment (33.0 ± 3.3 a.u.; *p *< 0.01; *n *= 4; Figure 2a and 2b). The morphological changes of the microglia and astrocytes were suppressed by ropivacaine treatment (Figure 2a). On the contralateral side, the immunoreactivities for both CD11b and GFAP were slightly, but significantly, increased (Figure 2), as described in previous reports [32,33]. These increases in immunoreactivity were also decreased after ropivacaine treatment (Figure 2).

### Effects of blockade of NGF action on the ropivacaine-induced suppression of activated spinal glial cells

**Figure 3:**
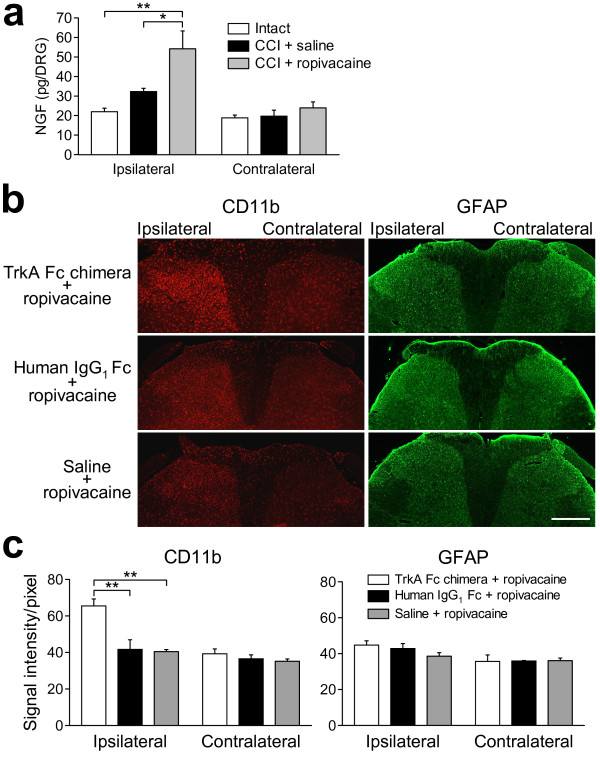
**Effects of the TrkA-Fc chimera on the ropivacaine-induced suppression of activated glial cells in CCI rats**. (a) The NGF contents on the contralateral and ipsilateral sides were measured in control intact rats (white columns), CCI rats treated with epidural saline (black columns) and CCI rats treated with epidural ropivacaine (grey columns). *p < 0.05 and **p < 0.01 by Tukey-Kramer's multiple comparison test (*n *= 8-9). (b) Representative images of immunohistochemical staining for CD11b (a microglial activation marker) and GFAP (an astrocyte activation marker) in the L4 spinal dorsal horn. L4 spinal cords were obtained from CCI rats treated with the TrkA-Fc chimera and ropivacaine, CCI rats treated with the control IgG_1_-Fc protein and ropivacaine, and CCI rats treated with saline and ropivacaine at day 10 after CCI. Scale bar, 200 μm. (c) Densitometric quantification of the CD11b and GFAP immunoreactivities. **p < 0.01 by Tukey-Kramer's multiple comparison test (*n *= 4).

As previously described [30], the NGF content in the lumbar fourth (L4) DRG on the ipsilateral, but not contralateral, side of CCI rats was significantly increased by ropivacaine treatment (*p *< 0.01 compared with control intact rats and *p *< 0.05 compared with CCI rats with saline treatment; *n *= 8-9; Figure 3a). To clarify the involvement of NGF in the inhibitory effect of ropivacaine on the activated glial cells, we blocked the action of NGF in CCI rats with ropivacaine treatment and examined its effect on glial activation at day 10. NGF was specifically blocked with a recombinant rat TrkA-Fc chimera by sequestration of endogenous NGF. In CCI rats with ropivacaine, the suppression of the morphological change in microglia after CCI was blocked by prior administration of the TrkA-Fc chimera (Figure 3b). In contrast, the suppression of the morphological change of astrocytes was not affected by the blockade of NGF action (Figure 3b). Densitometric analysis revealed that the inhibitory effect of ropivacaine on the immunoreactivity for CD11b on the ipsilateral side was significantly blocked by treatment with the TrkA-Fc chimera compared with treatment with the control IgG_1_-Fc protein or saline at day 10 (65.5 ± 3.8 a.u. for TrkA-Fc treatment, 41.7 ± 5.3 a.u. for IgG_1_-Fc treatment and 40.4 ± 1.2 a.u. for saline treatment; *p *< 0.01; *n *= 4; Figure 3c). In addition, the inhibitory effect of ropivacaine on the immunoreactivity for GFAP on the ipsilateral side was not significantly blocked by treatment with the TrkA-Fc chimera at day 10 (44.8 ± 2.4 a.u. for TrkA-Fc treatment, 42.8 ± 2.8 a.u. for IgG_1_-Fc treatment and 38.6 ± 2.0 a.u. for saline treatment; *n *= 4; Figure 3c). On the contralateral side, the immunoreactivities for both CD11b and GFAP were unchanged among the CCI rats with each treatment (Figure 3b and 3c).

### Effect of blockade of NGF action on the ropivacaine-induced analgesia

**Figure 4:**
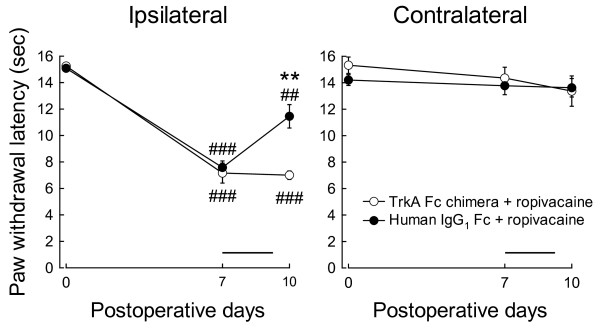
**Effect of the TrkA-Fc chimera on the analgesic effect of ropivacaine in CCI rats**. The latencies for paw withdrawal on the ipsilateral (left panel) and contralateral (right panel) sides in response to thermal stimuli in CCI rats were evaluated. Ropivacaine and the TrkA-Fc chimera (open circles) or control IgG_1_-Fc protein (closed circles) were epidurally administered once daily from days 7 to 9, as indicated by the horizontal bars. **p < 0.01 compared with the values in CCI rats treated with the IgG_1_-Fc protein on the corresponding days by an unpaired *t*-test (*n *= 5). ^##^*p *< 0.01 and ^###^*p *< 0.001 compared with the values on the preoperative day by Dunnett's multiple comparison test (*n *= 5).

We further examined the effect of the TrkA-IgG chimera on the ropivacaine-induced analgesia in CCI rats. Prior administration of the TrkA-IgG chimera significantly inhibited the ropivacaine-induced alleviation of the thermal hyperalgesia in CCI rats on day 10 compared with administration of the control IgG_1_-Fc protein (7.0 ± 0.3 s for TrkA-Fc treatment and 11.5 ± 0.9 s for IgG_1_-Fc treatment; *p *< 0.01; *n *= 5; Figure 4). The treatments with the control IgG_1_-Fc protein and TrkA-Fc chimera did not affect the contralateral paw withdrawal (13.4 ± 1.1 s for TrkA-Fc treatment and 13.6 ± 0.7 s for IgG_1_-Fc treatment; *n *= 5; Figure 4). However, chronic intrathecal administration of NGF (3 μg/day) from days 7 to 14 in CCI rats did not relieve the thermal hyperalgesia on day 14 after CCI (8.0 ± 0.3 s for NGF treatment and 8.9 ± 0.6 s for vehicle treatment; *n *= 3-4). Even a high dose of NGF (12 μg/day) could not ameliorate the thermal hyperalgesia (7.5 ± 1.0 s at day 14; *n *= 2).

## Discussion

In this study, we have shown for the first time that repetitive epidural administration of the local anesthetic ropivacaine suppressed both activated microglia and astrocytes concurrently with alleviation of thermal hyperalgesia in a rat model of neuropathic pain. Furthermore, upregulated NGF in the injured DRG was involved in the suppression of activated microglia, but not astrocytes, and may contribute to the prolonged analgesic effect of ropivacaine.

In recent years, microglia have been increasingly receiving much attention due to their potential as therapeutic targets for intractable pain. Many compounds that modify microglial function successfully alleviate neuropathic pain [15,16,18]. Consistently, the present study showed that ropivacaine suppressed activated microglia and relieved neuropathic pain via the upregulation of NGF expression in the DRG, suggesting NGF-dependent microglial inhibition by ropivacaine. Thus, NGF upregulated in the DRG by ropivacaine may act on microglia through transport along the DRG axons and be released into the spinal cord. Under some conditions, NGF receptors, TrkA and p75 neurotrophin receptors, are reported to be expressed in microglia and to be involved in their function [34-37]. Alternatively, NGF may diminish the injury-induced expression of activators for microglia in DRG neurons. It is well known that NGF is a key neurotrophic factor for maintaining the function of a subpopulation of DRG neurons [38] and restores damaged functions of the primary afferents in a wide range of disorders [22,23,39-41]. Upon nerve injury, DRG neurons begin to release CCL2 in the dorsal spinal cord, leading to microglial activation [11,42,43]. On the contrary, interleukin (IL)-10, an anti-inflammatory cytokine, is also increased after nerve injury [44]. IL-10 suppresses the p38 MAPK activation and tumor necrosis factor-α expression in microglia activated by lipopolysaccharide [45]. Therefore, NGF may modulate the expression of injury-induced cytokines in the DRG neurons, and thereby indirectly prevent the activation of microglia.

The ropivacaine-induced analgesia was abolished by NGF blockade, whereas NGF by itself, even at quite a high dose, could not relieve neuropathic pain. While it is well established that NGF has a hyperalgesic action in the periphery [19], the roles of NGF in nociceptive modulation in the spinal cord are still controversial. It was reported that intrathecal administration of NGF induces thermal hyperalgesia in intact rats [46]. On the contrary, other reports have indicated that NGF may be rather beneficial for the treatment of neuropathic pain when administered intrathecally [20,21,26]. Although the reason for the discrepancy in the results is not yet understood, the present study implies that ropivacaine could be a feasible lead compound for local upregulation of NGF in the injured DRG. Although we cannot speculate at present that all local anesthetics have an NGF-upregulation effect similar to ropivacaine, further studies using other drugs seem to be warranted because other local anesthetics, including butamben [47], bupivacaine [3,48] and lidocaine [4,5,7], have similar long-term analgesic effects to ropivacaine. In the present study, ropivacaine dose-dependently reduced the pain-related behavior. At all concentrations of ropivacaine examined, the rats showed transient motor paralysis after administration of ropivacaine. This dose-dependency could be interpreted as an analgesic effect of ropivacaine that is related to voltage-gated sodium channel blockade. However, the experiment examining the prolonged effect of ropivacaine showed that the analgesic effect of 0.2% ropivacaine continued for at least 2 days after the recovery from the hyperalgesia that was established at day 10 by the repeated daily injections. Therefore, the prolonged effect of ropivacaine in the present study seems to be beyond the transient voltage-gated sodium channel blockade. In addition to voltage-gated sodium channel blockade, local anesthetics are known to affect many different molecules including G protein-coupled receptors and immune cells [2]. Therefore, molecular targets other than voltage-gated sodium channels may contribute to the NGF upregulation and prolonged analgesic effect, although the channel blockade may initiate these processes.

Astrocytes have also been shown to play an important role in neuropathic pain [11,13], although much less is known about the molecular basis. Upon activation, astrocytes increase their synthesis of inflammatory factors similar to microglia [11,12], and compounds that inhibit activated astrocytes have been shown to attenuate neuropathic pain [12,17]. Consistent with these findings, activated astrocytes were also inhibited by ropivacaine treatment. However, the ropivacaine-induced suppression of activated astrocytes was not prevented by blockade of NGF action. Therefore, the inhibitory effect of ropivacaine on astrocyte activation seems to be NGF-independent, in clear contrast to the effect on microglia. It still remains to be elucidated whether the inhibition of astrocyte activation is required for the analgesic effect of ropivacaine.

## Conclusions

The present study has shown that ropivacaine suppressed activated microglia and astrocytes in the spinal dorsal horn in a neuropathic pain state, concomitantly with the alleviation of pain. The suppression of activated microglia and the analgesic effect of ropivacaine were mediated by NGF signaling. These results suggest that epidural local anesthetics, including ropivacaine, may represent a new approach for glial cell inhibition and, therefore, therapeutic strategies for neuropathic pain.

## Methods

### Experimental animals

All experimental procedures were approved by the Nippon Medical School Animal Care and Use Committee (Approval number 21-036) and carried out in accordance with the guidelines of the International Association for the Study of Pain [49]. Male Sprague-Dawley rats (weighing 220-300 g at the time of surgery) were used for all experiments. All surgical procedures were performed on rats that were deeply anesthetized with sodium pentobarbital (50 mg/kg, intraperitoneally). The rats were singly housed under a 14-h light cycle and received food and water *ad libitum*.

### Production of a neuropathic pain model

To induce neuropathic pain, the rats were subjected to CCI essentially as described previously [50], except that 4-0 silk thread was used instead of chromic gut. The left (ipsilateral) sciatic nerve was exposed at the mid-thigh level and four 4-0 silk threads were loosely ligated around the nerve at intervals of approximately 1 mm. The incision was closed with a 4-0 silk suture. The right (contralateral) sciatic nerve was left intact. Control intact rats were housed without any surgery until tissue sampling.

### Epidural catheterization

For epidural administration of drugs, a polyethylene catheter (PE-10; Natsume, Tokyo, Japan) was implanted into the epidural space at day 6 after CCI, as previously described [31]. Briefly, a catheter filled with saline was gently introduced from the base of the lumbar fifth (L5) spinous process into the lumbar epidural space to a length of 2 cm, so that its tip was located at the L3-L4 level. The catheter was then flushed with 100 μl of saline to ensure that was no leakage from the epidural space. The catheter was tied with a loose knot at a level between the L5 and lumbar sixth (L6) vertebrae and secured to superficial lumbar muscles. The catheter was then tunneled subcutaneously to the surface of the neck skin and fixed to its fascia. Catheter-implanted rats without obvious movement disturbances, such as paralysis, were used in subsequent experiments. After completion of the experimental series, we confirmed that the tip of the catheter was appropriately located in the epidural space at the L3-L4 level. Furthermore, in preliminary experiments, we confirmed that dye injected through the catheter was reliably delivered to the epidural, but not the subdural, space around the L3-L4 level.

### Epidural administration of drugs

One hundred microliters of 0.1%, 0.2% or 0.75% ropivacaine (AstraZeneca, Osaka, Japan) or saline was slowly injected for 30 s through the epidural catheter, followed by 20 μl of saline. Except for the experiment examining the dose-dependent effects of ropivacaine, 0.2% ropivacaine was used in all experiments. As we reported previously [27], this dose of ropivacaine caused the rats to show transient motor paralysis immediately after the administration, but no other systemic effects during the experiments. Ropivacaine was administered once daily from days 7 to 13 after CCI. In the experiment to examine the duration of ropivacaine analgesia, ropivacaine was administered once daily from days 7 to 10. To block the actions of NGF, a recombinant rat TrkA-Fc chimera (Recombinant Rat TrkA-Fc Chimera; R&D Systems, Minneapolis, MN) was epidurally injected at 1 h before ropivacaine administration from days 7 to 9 after CCI. As a control, a recombinant human IgG_1_-Fc protein (R&D Systems) or saline was injected. Both recombinant proteins were dissolved in sterile saline (0.1 μg/μl) and were epidurally administered at a dose of 50 μl, followed by 20 μl of saline.

### Intrathecal catheterization and NGF administration

An intrathecal catheter was inserted on the same day as the CCI surgery, according to a previously described method [51]. Briefly, a polyethylene catheter (PE-10) was inserted into the subarachnoid space through a slit between the L5 and L6 vertebrae and advanced by 2 cm to reach the lumbar enlargement of the spinal cord. The insertion of the catheter into the subarachnoid space was verified by cautious aspiration of cerebrospinal fluid. Rats displaying obvious hind limb paralysis were excluded from the study. At 7 days after CCI and catheterization, the free extremity of the catheter was connected to an osmotic pump (model 2001; Alzet, Cupertino, CA) filled with 125 or 500 ng/μl of rat recombinant β-NGF (Sigma-Aldrich, St. Louis, MO) dissolved in saline containing 1 μg/μl of rat serum albumin (Sigma-Aldrich). Saline with albumin was used as a control. The drug was pumped out at a rate of 1 μl/h for 7 days.

### Behavioral test

Thermal hyperalgesia was assessed using a Plantar Test (Ugo Basile, Varese, Italy) on day 0 before and on days 3, 7, 10 and 13 after CCI. From days 7 to 13, a behavioral test was performed before drug administration. Briefly, each rat was placed on a glass plate with a radiant heat generator (a 55-W halogen reflector bulb) underneath. After an acclimation period, radiant heat was applied to either the contralateral or ipsilateral hindpaw pad independently. The latency of paw withdrawal from the thermal stimuli was measured twice at 5-min intervals and the average value was adopted. Since our previous study revealed that ropivacaine was less effective at treating mechanical allodynia [30], we focused on thermal hyperalgesia to examine the behavioral effect of ropivacaine.

### Two-site enzyme immunoassay for NGF

The NGF protein concentrations were measured by a two-site enzyme immunoassay (EIA) [52]. At 14 days after CCI, the portions of the ipsilateral and contralateral sides of the L4 DRG were immediately dissected, frozen in liquid nitrogen and stored at -80°C. Each tissue was homogenized using a Polytron homogenizer (NITI-ON, Chiba, Japan) in 500 μl of homogenization buffer (50 mM Tris-HCl pH 7.5, 0.5 M NaCl, 0.3% Triton X-100) containing protease inhibitors (Complete Mini; Roche Diagnostics, Mannheim, Germany). The homogenate was centrifuged at 12,000 rpm for 30 min at 4°C and the supernatant was used for NGF measurement. EIA titer plates (FluoroNunc Module Plates; Nunc, Roskilde, Denmark) were coated with a primary polyclonal antibody against NGF (20 ng/well; Promega, Madison, WI) overnight and then blocked with EIA buffer (50 mM Tris-HCl pH 7.5, 0.5 M NaCl, 0.3% Triton X-100, 0.4% bovine albumin, 0.4% gelatin) at 4°C for more than 3 h. Next, 100 μl of each tissue extract or 1-1000 pg of NGF-β standard (recombinant human NGF-β; Chemicon, Temecula, CA) in EIA buffer was placed in each well and the plates were incubated for 7 h at room temperature. After three washes with EIA buffer without bovine serum albumin, 100 μl of an anti-NGF monoclonal antibody conjugated to β-galactosidase (5 μg/ml; Boehringer Mannheim, Mannheim, Germany) in EIA buffer was added to each well and the plates were incubated for 12-18 h at room temperature. After a subsequent incubation with 200 μM 4-methylumbelliferyl β-D-galactoside (Sigma-Aldrich) in 50 mM sodium phosphate buffer (pH 7.3) containing 10 mM MgCl_2 _for 24 h in the dark at room temperature, the resulting fluorescent products were measured using a Spectrafluor Plus Microplate Reader (Tecan, Salzburg, Austria) with excitation at 355 nm and emission at 460 nm.

### Immunohistochemistry

The rats were perfused transcardially with phosphate-buffered saline (PBS; pH 7.2) followed by freshly prepared 4% paraformaldehyde in PBS (pH 7.4). The L4 spinal cord was dissected out, post-fixed in the same fixative at 4°C overnight and then cryoprotected in 20% sucrose in PBS at 4°C overnight. Subsequently, the spinal cord was rapidly frozen in dry ice/acetone and cut into 16-μm transverse sections using a cryostat (Leica Microsystems, Wetzlar, Germany). The sections were preincubated in PBS containing 5% normal donkey serum and 0.2% Triton X-100 at room temperature for 30 min and then incubated with a mouse monoclonal anti-CD11b antibody (OX-42, a microglial activation marker; 1:1000 dilution; Serotec, Oxford, UK) or a rabbit polyclonal anti-GFAP antibody (1:300 dilution; Dako Cytomation, Glostrup, Denmark) at 4°C overnight. The specificity of the anti-GFAP antibody was shown in a previous study [53]. After washing with PBS, the sections were incubated with an Alexa Fluor 594-conjugated anti-mouse IgG antibody (1:1000 dilution; Life Technologies, Carlsbad, CA) or an Alexa Fluor 488-conjugated anti-rabbit IgG antibody (1:1000 dilution; Life Technologies). Images were captured using a high-resolution digital camera (DP71; Olympus, Tokyo, Japan) equipped with a computer. To quantify the immunofluorescence intensity, two sections (minimum separation, 100 μm) from the L4 spinal cord were examined per animal. The dorsal horn was densitometrically analyzed using a computerized image analysis system (ImageJ; NIH, Bethesda, MA). Data are presented as the average signal value in arbitrary units (a.u.) from 0 to 255 per pixel. All of the quantitative analyses were performed by a blinded tester.

### Statistical analysis

Values are expressed as means ± SEM. Differences in the paw withdrawal latencies before and after surgery or ropivacaine administration, and on the ipsilateral and contralateral sides of the hindpaws on the corresponding day were analyzed by Dunnett's multiple comparison test and an unpaired *t*-test, respectively. The Tukey-Kramer multiple comparison test was used to compare the NGF contents and immunofluorescence intensities of CD11b and GFAP among control intact rats, CCI rats and CCI rats with ropivacaine treatment and among CCI rats treated with the TrkA-Fc chimera, control IgG_1_-Fc protein and saline. Values of *p *< 0.05 were considered to indicate statistical significance.

## List of abbreviations

CCI: chronic constrictive injury; DRG: dorsal root ganglion; EIA: enzyme immunoassay; IL: interleukin; L3: lumbar third; L4: lumbar fourth; L5: lumbar fifth; L6: lumbar sixth; NGF: nerve growth factor.

## Competing interests

The authors declare that they have no competing interests.

## Authors' contributions

ST and AS participated in all aspects of the study and in the writing of the manuscript. YI participated in the enzyme immunoassay analyses. AS and HS participated in the study design and supervision, and in the writing of the manuscript. All the authors read and approved the final manuscript.
